# A Peculiar Case—No. 5

**Published:** 1862-11

**Authors:** 


### A PECULIAR CASE—No. 5.

Oct. 8, 1862.—Mrs. B. called to-day, and presented the following rather singular case. The first inferior right molar has been largely decayed upon the posterior proximal surface, the pulp exposed, though it had not ached; it was found in this condition a few weeks ago by one of our best operators, the cavity was cleansed out, and an application made to the pulp to improve its condition. This treatment occupied three or four days, at the expiration of which time the tooth was prepared for filling: the decayed cavity was filled with gold. There was no pain at the time of filling, and none subsequently, till within perhaps ten days, and painful then only, when heat or cold was applied to the tooth, then excruciating pain was experienced; the only variation from a normal sensation under percussion, or pressure upon the tooth, was about the same as that manifested when there is a slight periostial irritation; rather an invitation to pressure than otherwise. Water at about 65° thrown upon it produced intense pain; my impression was, that the pulp was not dead. There was a small decay in the central depression upon the grinding surface of the tooth; in order to ascertain the true condition of affairs, I drilled through this into the pulp chamber, where I found no nerve, and no secretion; there were some nodules of osteo-dentine, filling up the principal part of the chamber; there was not the slightest sensitiveness.

Upon the chamber being perforated, the symptom referred to entirely disappeared, so that ice water could be thrown upon the tooth freely in a moment after the perforation, without producing the slightest pain, and the former sensation under percussion or pressure was wholly relieved.

Upon the perforation of the chamber there was no odor apparent, the chamber and canals were cleansed out, and fibres of flax moistened with creosote packed in the bottom, and the whole then well filled with gold, effecting a complete remedy of the symptoms.

The filling in the posterior surface of the tooth was not disturbed, as it was a large one, and a good one, and that was not the most convenient way of entrance into the chamber.

Now, there are two or three things about this case we should like to know ; in the first place, since there was no pulp, and no periostitus, what was the cause of the pain, under changes of temperature. The fact of there being no secretion in the cavity, still adds to the difficulty, and the absence of odor still adds a little more in that direction.

The only suggestion we will venture now, explanatory of the phenomenon is, that perhaps an odorless gas was generated in the chamber, and that heat communicated to that through the gold filling caused its expansion, and consequently pressure, through the foramen of the fang to the parts beneath ; and cold would produce a contraction, and induce pain by operating in the opposite direction. I don't *know* any thing about it ; will somebody please explain. May not such cases be far more frequent than we suppose. T.

